# Aperiodicity in Mouse CA1 and DG Power Spectra

**DOI:** 10.1523/ENEURO.0136-25.2026

**Published:** 2026-03-25

**Authors:** Gustav Kühn, Hussin El Rashidy, Federico Calegari, Shonali Dhingra

**Affiliations:** ^1^Center for Regenerative Therapies TU Dresden, Technische Universität Dresden, Dresden 01307, Germany; ^2^Faculty of Computer Sciences, Technische Universität Dresden, Dresden 01062, Germany

**Keywords:** aperiodicity, CA1, dentate gyrus, electrophysiology, hippocampus, local field potential

## Abstract

Rodent hippocampal power spectra comprise periodic and aperiodic components. The periodic components (brain rhythms) contain information about the behavioral or cognitive state of the animal. The aperiodic components are rarely studied, and their functionality is not well understood, though they have shown to be correlated with animal's age or the excitation–inhibition ratio of the brain region. To study these components in the mouse hippocampus, we modified the existing open-source FOOOF toolbox, which was originally optimized for EEG data. First, using simulated data, we show that our modifications decrease the error in assessment of the low frequency periodic components from 3 to 0.1%. Second, using tetrode electrophysiological signals from adult males, we compare the aperiodic activity within mice hippocampal subregions, CA1 and dentate gyrus (DG). Our optimization of FOOOF improved the aperiodic assessment errors by ∼50% and were critical in making the first assessment of the aperiodic components in these brain regions. Our results show significantly larger aperiodic exponents and knee frequencies in the DG than in CA1, which have been proposed to correlate with lower excitation and shorter timescales. Our work highlights the subtle differences in electrophysiology field potentials between hippocampal subregions and presents the improvements needed in the existing open-source toolbox to be able to see such differences.

## Significance Statement

Electrical brain signals comprise neuronal spiking activity and voltage fluctuations arising from extrasomatic and transmembrane processes, termed local field potentials (LFPs). The LFP comprises periodic brain rhythms overlaid on aperiodic background. Though spiking activity and brain rhythms have been under investigation for decades, the aperiodicity in these signals was often cast aside as “noise,” due to its ubiquitous nature. Our work estimates the aperiodic nature of rodent hippocampal signals for the first time, which suggests different neuronal timescales between hippocampal subregions. Our study will be important to assess changes in periodic and aperiodic parameters with hippocampal cognitive states, such as during hippocampal memory tasks, and sleep states.

## Introduction

In vivo electrophysiology (ephys) provides wide-band neural signals (direct current to 40 kHz), containing action potentials and other membrane potential-derived fluctuations, termed as local field potential (LFP), for cortical and deeper brain regions such as the hippocampus. Multiple synaptic and transmembrane processes in a small neural volume synergistically contribute to the LFP, which is correlated with various sensation, perception, and cognitive processes (Einevoll et al., 2013). On the other hand, EEG and ECoG also provide spatiotemporally smoothed versions of cortical LFP, integrated over an area of 10 cm^2^ or more (Buzsáki et al., 2012). Though LFP from ephys signals provides a more detailed view of the local neuronal environment, being more invasive than EEG makes it less commonly used in cortical primate studies, but frequently employed in hippocampal rodent studies.

The wide-band LFP signals contain oscillatory activity overlaid on top of aperiodic background. Neural oscillations are prominent features of rhythmic brain activity, which are thought to relate to population synchrony and coordination of neural activity at the population level (Buzsáki and Draguhn, 2004; Donoghue et al., 2022; Gerster et al., 2022; Kennedy et al., 2022). The most prevalent of these brain rhythms in the rodent hippocampus include theta (4–12 Hz) and slow gamma (sgamma, 25–50 Hz; Leung, 1998). Along with these brain rhythms, the power distribution at various frequencies follows a 1/*f^n^* statistics, commonly attributed to various extrasomatic and extracellular sources (Buzsáki et al., 2012). This aperiodic roll-off with frequency in these signals is occasionally cast aside as “noise,” due to its ubiquitous nature. Investigation of the aperiodic components has recently gained considerable interest, and the exponent *n* has been shown to change with task demands, age, and disease, among other correlations (Nunez, 1981; Buzsáki et al., 2012, 2013; Gerster et al., 2022). Though, to the best of our knowledge, complete estimation of the aperiodic components in the rodent hippocampus has never been reported.

Traditionally, the most commonly used method to estimate parameters of oscillatory activity in ephys data has been bandpass filtering (Bokil et al., 2010). Due to the abovementioned aperiodic activity, applying narrowband filtering can lead to nonzero power in the detection bands (Donoghue et al., 2020a; Gerster et al., 2022). This is sometimes avoided by broader band filtering and normalization by the average broadband power (Safaryan and Mehta, 2021), which could still lead to biased estimates of power in a certain narrow band, and frequency of maximum power in such a band, especially for lower frequency bands (Extended Data Fig. 1-1*a*).

Recently, there has been a big thrust in determining the aperiodic components of the neural signals to get better estimates of the periodic components (Wilson et al., 2022; Donoghue et al., 2024) and correlate them with behavioral or cognitive measures, such as mentioned above. A recently developed method, termed FOOOF, is currently widely used to separate out the rhythmic neural oscillations from the aperiodic or arrhythmic part of the spectrum (Donoghue et al., 2020b). This analytical method has mostly been used for EEG and ECoG studies in humans and macaques, with only very few reports so far using it to analyze hippocampal ephys recordings in rodents (Gao et al., 2017; Kala et al., 2023; Leemburg et al., 2024).

To be able to reliably use FOOOF for hippocampal ephys signals, we made vital modifications to its assessment procedures of aperiodic and periodic components (see Results and Materials and Methods for details). In brief, we added three new aperiodic fitting models more suited for ephys signals, restricted the detection of the periodic components to be within hippocampus-relevant frequency bands, and iteratively implemented these steps to minimize errors. We then used our modified toolbox to analyze recordings in the CA1 and dentate gyrus (DG) of the mouse hippocampus.

## Materials and Methods

### Experimental design and statistical analyses

#### Subjects

Three adult male C57BL/6JRj mice (Janvier Labs; ∼3–4 months old at the time of surgical implantation) were individually housed on a 12 h dark/light (reverse) cycle. Animals were given ad libitum food and water. All experimental procedures were approved by local authorities and compiled with all relevant ethical regulations (TVV 49/2022).

#### Animal handling and surgery

The mice were handled for ∼15 d to get them used to the room and being gently restrained within and between the hands of the experimenters. The animals that stayed calm, yet curious, during these conditions were then considered for implantation. The mice were implanted with 2–3 g ShuttleDrive from Open Ephys (Voigts et al., 2020), with 12–14 individually adjustable tetrodes (13 µm nichrome wires) positioned unilaterally on the right hemisphere over dorsal CA1 (−2.0 mm A.P., 1.7 mm M.L. relative to bregma). Surgery was performed under isoflurane anesthesia and heart rate, breathing rate, and body temperature were continuously monitored. Analgesia was achieved by using metamizol (200 mg/kg, s.c.), buprenovet (0.1 mg/kg, s.c.), and Xylocaine gel (2%, topical). One 1.6-mm-diameter craniotomy was drilled using a handheld drill. Dura mater was removed, and the ShuttleDrive was lowered until the cannula is level with the skull surface. The implant was anchored to the skull with six skull screws and dental cement. One parietal and one occipital skull screw was used as ground for recording. Mice were administered 150 mg metamizol in 100 ml drinking water (200 mg/kg) for 7 d during recovery.

#### Environment and animal behavior

All experiments were conducted inside a 1.5 m cubic EMF-shielded enclosure, with only red lights to maintain the reverse light cycle. The animals ran in an open-field environment (OFE), a 50 cm square box, while they foraged for randomly scattered chocolate sprinkles. The base of the OFE box was covered with a white uniform smooth mat, which was wiped clean at the end of each session with ethanol to minimize scents between days. The animal's behavior was captured with a 55 Hz color Chameleon3 FLIR camera and synced with the neural data using Bonsai (Lopes et al., 2015).

#### Electrophysiology and neural signal acquisition

Neural signals were acquired using Intan recording systems (C3100, sampling rate 30 kHz) and visualized on Open Ephys GUI, using OpenEphys low-profile SPI headstage 64ch. The tetrodes were lowered gradually after surgery through the cortex into the hippocampus. The cortical data in Figure 2 is from tetrodes in somatosensory, parietal, or visual areas. Their position in the cortex is confirmed with the presence of up-down states. Positioning of the electrodes in CA1 and DG was confirmed through the presence of sharp-wave ripples (SWR) and dentate spikes during recordings and through histology after experiments (Extended Data Fig. 3-1*a,b*). The CA1-radiatum data in Extended Data Figure 3-1 is also from the same tetrodes after passing through CA1-pyramidale layer (same data labeled as CA1, where not specified), which was confirmed with SWR amplitude and polarity.

#### Preprocessing of neural data

A low-pass (8th order Butterworth) filter at 500 Hz was applied to the ephys signals and then downsampled to 1 kHz to be used as LFP traces. Application of this filter does not change the aperiodic properties of the signals until 400 Hz, as compared with original, unfiltered nondownsampled data (Extended Data Fig. 3-1*d*). The segments when the animal was moving below a speed of 5 cm/s for at least 450 ms were removed, to avoid any confounds in the periodic or aperiodic components with sleeping or quiescent states. In total, we included data from 21 sessions, with each ranging between 8.5 and 18 min.

**Figure 1.**
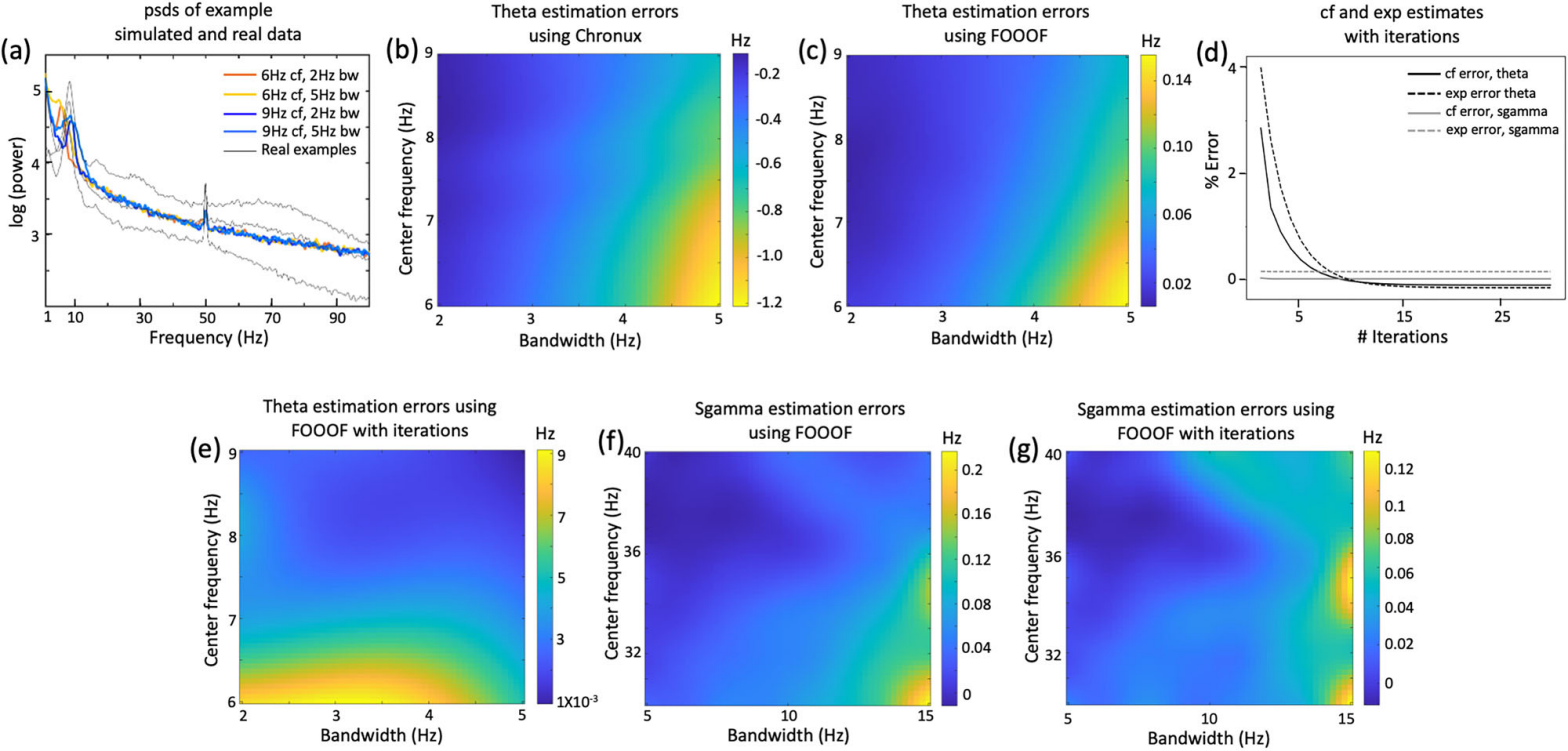
Better estimates of the periodic components in simulated electrophysiological signals. ***a***, psds of four example simulated hippocampal signals, with theta as a Gaussian peak with a center frequency (cf) varying between 6 and 9 Hz, bandwidth (bw) between 2 and 5 Hz, and power of ∼15 dB, with pink-noise exponent of 1.2. Examples of real rodent tetrode hippocampal signals are also shown for comparison of noise levels. ***b***, Errors in cf estimates of the simulated theta peak using Chronux toolbox, with varying bw and cf. This and further such plots were obtained by averaging over 20 initiations of simulated data and smoothed using a Gaussian filter with std of 4, unless specified. ***c***, Same as ***b*** but using FOOOF. ***d***, Percentage errors in cf and exponent estimates for simulated theta (cf = 6 Hz, bw = 5 Hz) and sgamma (cf = 30 Hz, bw = 15 Hz) peaks with iterative aperiodic and periodic estimation in FOOOF. ***e***, Same as ***c*** after 20 iterations in FOOOF. ***f***, ***g***, Same as ***c***, ***e*** but for simulated sgamma peaks. Further error estimations can be found in Extended Data Figure 1-1.

For each electrode, the power spectral density (psd) was calculated, using the Welch's method with a Hanning window size of 1.2 s (3 s for Fig. 1), nfft of 4,000, and 50% overlap. The electrode with the highest total power $\left[\sum_{1<F<495}\log_{10}\mathrm{psd}(F)\right]$ was chosen from each tetrode. Electronic noise peak at 50 Hz was removed from the psd by applying the “1exp” FOOOF fit in a small range (43–57 Hz) and subtracting the fitted Gaussian. The same procedure was applied to eliminate any harmonics of theta and 50 Hz noise.

For Extended Data Figure 1-1*b*, signals were bandpass filtered with a 4th order Butterworth filter (4–12 Hz), and the mean frequency of the resulting signal was estimate with MATLAB’s “meanfreq” function. For Chronux-related figures, theta-band power (4–12 Hz) was estimated using multitaper spectral analysis (mtspecgramc, TW = 2, *K* = 3) with 5 s windows and 1 s steps.

### Code/software

#### Simulated data

All the parameters for simulating the neural signals were chosen to emulate our tetrode ephys hippocampal signals. Time series were generated by applying frequency-domain filters to the FFT of Gaussian noise (noise-level adjusted to be consistent with our data; Fig. 1*a*), imposing a 1/*f^n^* background and a Gaussian-shaped bump at the desired center frequency (cf) and bandwidth (bw), across the full spectral range from 1 to 500 Hz; the modified spectra were mirrored to enforce Hermitian symmetry and then inverted to the time domain. An example script for the generation of simulated data is available in the Extended Data. For Figure 1, we added the 1/*f^n^* with *n* of 1.2 and put in an offset such that the power at 1 Hz is 60 dB. The exponent of 1.2 chosen here was slightly higher than the maximum for real DG data (∼1.1, median ∼0.7; Fig. 3*b*) to highlight the potential pitfalls in using FOOOF, especially when the oscillatory components are at lower frequencies. The theta peak was put in as a Gaussian peak with a cf of 6–9 Hz, maximum power of ∼6 dB above the aperiodic power at cf, and a standard deviation of 1–2.5 Hz (2–5 Hz bw). Similarly, to simulate peaks in the sgamma band, we put in a Gaussian peak with cf of 30–40 Hz, maximum power of ∼2 dB above the power at cf, and bw of 5–15 Hz bw. We also put in a 2.5 dB, 0.5 Hz bw noise peak centered at 50 Hz and another one centered at 150 Hz with the same parameters. For Figure 1*c–g*, the simulated time series was subjected to the same preprocessing steps, as mentioned above.

#### Equations for the aperiodic fits

Simplified forms of the equations are shown here for clarity, the exact forms can be found in the available code.

For 4–100 Hz and 4–200 Hz, log(power) L for each spectrum could be fit to the following:

1exp:

$$L=b-\log(\;f^{n}),$$

where *n* is the exponent across frequencies *f.*

flat + 1exp:

$$L=b-\log\left(1+{\left(\frac{\;f}{k}\right)}^{n}\right),$$

where *k* is the knee frequency in Hz and *n* is the exponent across frequencies *f*.

2exp:

$$L=b-\log\left({\left(\frac{\;f}{k}\right)}^{n_{1}}+{\left(\frac{\;f}{k}\right)}^{n_{2}}\right),$$

where *k* is the knee frequency in Hz and *n*_1_ *and n*_2_ are the exponents across frequencies *f*.

For direct comparison of the “2exp” fitting with “flat + 1exp” fitting, we put the following conditions on the aperiodic components after the fittings, only for plotting: if the absolute difference between the two exponents was <0.01, or if the reported “knee” frequency was >195 Hz, we changed the 1st exponent to be 0, 2nd exponent to be the as-obtained value, and the knee frequency to 4 Hz. This was done to define more biologically meaningful parameters from the as-obtained parameter values.

For 4–400 Hz fitting: We restricted the fitting to 400 Hz to eliminate roll-off artifacts:

2exp + flat:

$$L=b-\log\left({\left(\frac{\;f}{k}\right)}^{n_{1}}+{\left(\frac{\;f}{k}\right)}^{n_{2}}\right)+\;\log\left({\left(\frac{\;f}{k}\right)}^{n_{2}}+(k^{\prime })\right),$$

where *n*_1_ *and n*_2_ are the exponents across frequencies *f* with *n*_2_ > *n*_1_, *k* is the 1st knee, and (|*k*′/*n*_2_|+*k*) is the 2nd knee frequency, both in Hz.

3exp:

$$L=b-\log\left({\left(\frac{\;f}{k}\right)}^{-s_{2}}+{\left(\frac{\;f}{k}\right)}^{s_{3}}\right)+\;\log\left({\left(\frac{\;f}{k^{\prime }}\right)}^{-s_{1}}+{\left(\frac{\;f}{k^{\prime }}\right)}^{s_{2}}\right),$$

where *n*_1_ = *s*_1_ − *s*_2_, *n*_2_ = s_1_ + s_3_, and *n*_3_ = *s*_3_ − *s*_2_ are the three exponents, with *s*_1_, *s*_2_, *s*_3_ > 0 and *k* and *k*′ are the 1st and 2nd knee frequencies in Hz, respectively.

To avoid over-fitting until 400 Hz, we implemented step-wise curve-fitting, i.e., fit the data until 200 Hz with the “2exp” function, fixed the “1st exponent,” “1st knee frequency,” and starting power at 4 Hz, which might be biologically more relevant, and then refit the data. This allows the 2nd and 3rd exponent, and the 2nd knee, to be fit freely. This step-wise fitting prevents our mathematical models from messing up potentially biologically relevant parameters.

#### Detection of periodic components

We started both aperiodic and periodic assessment at 4 Hz to avoid delta rhythm, which can be inconsistent in the hippocampus, especially during active exploration (Fujisawa and Buzsáki, 2011; Mofleh and Kocsis, 2021). As done in the original FOOOF toolbox (Donoghue et al., 2020b), we processed each psd by subtracting the most suitable aperiodic fit from it resulting in a flattened spectrum. Thereafter, we used FOOOF's multi-Gaussian fitting method, but with restricting the peak detection process to fit only one peak per hippocampus-relevant oscillatory bands. This was done to avoid an individual brain rhythm being represented as a combination of multiple Gaussians, which posed a challenge for further analyses and interpretation. This additionally allowed us to define individual thresholds and restrictions for each oscillatory band. The parameters used in this process are outlined in Table 1. Note that this approach is different from extracting the highest Gaussian fit in each separate band after the fitting process, which is an alternative approach implemented in the FOOOF library.

**Table 1.** Parameters used to detect periodic components

Detection range (Hz)	Cf-bounds (Hz)	Peak-width limits (Hz)	Peak thresholds	Min. peak-height (log10(psd))
4–12 (theta)	5, 9.5	2, 5	1	0.1
19–48 (sgamma)	25, 40	5, 25	1	0.05
51–90 (fgamma)	52, 80	3, 35	1	0.08
100–200 (ripples)	125, 160	10, 30	1	0.05

The flattened psd was divided into hippocampus-relevant bands, and then a Gaussian peak was detected within individual bands. Different parameters were used within each band to optimize the peak detection for our electrophysiological data.

#### Errors estimation

**Figure 2.**
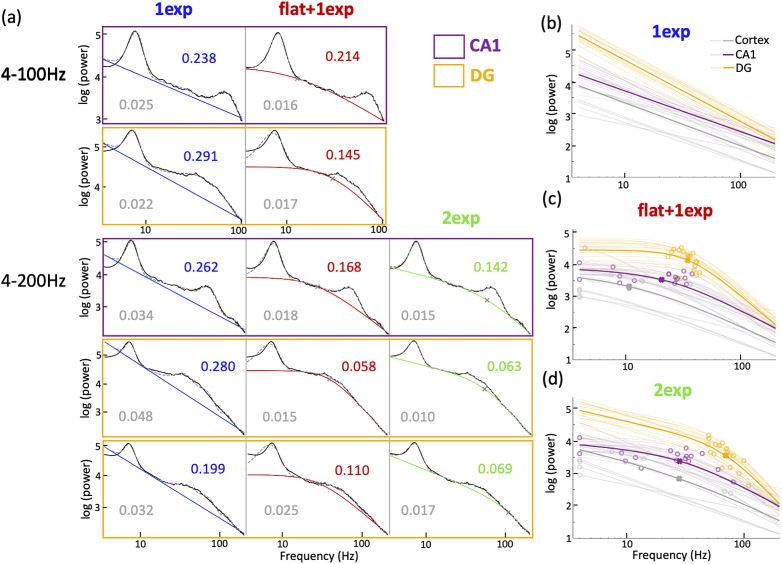
Aperiodic fits of electrophysiological signals between 4 and 200 Hz. ***a***, Examples of aperiodic fitting of mouse hippocampal CA1 (purple) and DG (yellow) electrophysiological signals using “1exp” (blue), “flat + 1exp” (red) and “2exp” (green), between 4–100 Hz (rows 1–2) or 4–200 Hz (rows 3–5). Aperiodic fit errors are shown in color on top right for respective fitting methods, and the corresponding full model fit errors are show in gray on bottom left. Rows 1 and 3 show the same dataset, as do Rows 2 and 4. ***b***, Aperiodic fits using “1exp” of all data from DG (yellow, *n* = 17), CA1 (purple, *n* = 17), and cortex (gray, *n* = 10). The average of all fits is shown in bold. Welch's *t* test, exponent: *p*(DG|CA1) = 6.82 × 10^−10^, *p*(CA1|Cortex) = 0.601. ***c***, Same as ***b*** but using “flat + 1exp,” with the respective “knee” frequencies as open circles and the average “knee” frequency as crosses. Welch's *t* test, exponent: *p*(DG|CA1) = 1.46 × 10^−8^, *p*(CA1|Cortex) = 0.231, “knee” frequency: *p*(DG|CA1) = 1.97 × 10^−4^, *p*(CA1|Cortex) = 0.0311. ***d***, Same as ***c*** but using “2exp”. Welch's *t* test, 2nd exponent: *p*(DG|CA1) = 1.5 × 10^−11^, *p*(CA1|Cortex) = 0.582, 1st exponent: *p*(DG|CA1) = 3.68 × 10^−10^, *p*(CA1|Cortex) = 0.298, “knee” frequency: *p*(DG|CA1) = 4.63 × 10^−7^, *p*(CA1|Cortex) = 0.989.

To quantify the goodness of our fittings, we used two kinds of error estimates as follows:

1. Full model fit error: This was calculated to quantify the fittings of the combined aperiodic and periodic components. To do this, we took the mean absolute difference of the full model fit (aperiodic fit + Gaussian fits) to our data and the psd, across the frequency points under consideration. This is identical to the “error of the fit” computed by the original FOOOF algorithm.
2. Aperiodic fit error: Since the full model error depends on both aperiodic and periodic fits, this can allow the periodic component to compensate for bad aperiodic fits. To quantify the fittings of the aperiodic components, we took the mean absolute difference of the psd of our data and full aperiodic fits (as shown in Fig. 2*b–d*), across the frequency points under consideration.

As expected, the full model fit errors are only marginally affected by the fitting modes, as flattening out the spectrum using the aperiodic components already makes the periodic fitting quite good.

#### Bayesian information criterion calculations

Bayesian information criterion (BIC) scores were calculated for all the fitting models using the following equations:

$$\mathrm{BIC}=n\ln\;(\sigma^{2})+k\;\ln(n),$$

where *n* is the number of frequency bins, *k* is the number of free parameters, and *σ*^2^ is the mean of the mean square of the ap error for each model.

#### Code accessibility

The code/software described in the paper is freely available online at (https://github.com/huRashidy/LFP_FOOOF/tree/main) and as Extended Data. We successfully implemented these codes on multiple computers using Windows operating system.

10.1523/ENEURO.0136-25.2026.d1 Extended Data 1 Python code for Fitting algorithm compatible with FOOOF 1.1.0 and example script for generation of simulated time-series. Download Extended Data 1, ZIP file.

## Results

### Improved assessment of periodic parameters

Over the years, several methods have been used to estimate the parameters of various periodic components in ephys LFP. Most of these methods can be categorized into one of the two methodologies of either working in the time-domain or in the frequency-domain. For the methods working in the time-domain, the time series data is subjected to either narrow (Ravassard et al., 2013) or broad (Belluscio et al., 2012) bandpass filtering, further using the filtered data to estimate the center frequency and power in a specific frequency band. In the more recent papers, with the understanding of the existence of 1/*f^n^* statistics in the neural data, these estimates were made on the data in the frequency domain (Bokil et al., 2010; Safaryan and Mehta, 2021; Strüber et al., 2022).

To test the efficacy of the commonly used time-domain and frequency-domain methods in estimating the center frequency of hippocampal theta rhythm, we simulated a noisy time series with a Gaussian theta peak with a center frequency (cf) of 6–9 Hz and a standard deviation of 1–2.5 Hz [2–5 Hz bandwidth (bw)]. To insert an aperiodic component into the same, we added a “pink noise-like” spectrum into it, which is simply a 1/*f^n^* noise, with an exponent of 1.2. For a realistic simulation, we also added a noise peak at 50 Hz (see Materials and Methods for more details; Fig. 1*a*). The parameters for simulations were chosen based on the medians in real tetrode ephys data from the mouse hippocampus.

10.1523/ENEURO.0136-25.2026.f1-1 Figure 1-1 **Better estimates of the periodic components in simulated electrophysiological signals** (a) Errors in cf estimates of the simulated theta peak with pink-noise exponent of 1.2, using band-pass filtering. (b) Errors in cf estimates of the simulated theta peak with pink-noise exponent of 0, using Chronux. This was smoothed using a Gaussian filter with std of 1. (c) Average errors in cf estimates using FOOOF, of the simulated theta peak (with bw of 3.5 Hz), with varying exponent and cf. (d) Flattened simulated and assessed theta Gaussian peak (cf = 6 Hz, bw = 5 Hz, exponent = 1.2) using FOOOF, without iterations. (e) Same as (d) but after 20 iterations. (f) Removal of noise peaks during preprocessing does not significantly affect the estimated aperiodic parameters. Download Figure 1-1, TIF file.

Using both narrow bandpass filtering (in time-domain) and Chronux toolbox (in frequency-domain) led to 15–20% error in the cf estimates of the theta Gaussian peak (see Materials and Methods; Fig. 1*b*, Extended Data Fig. 1-1*a*). These estimates were comparably erroneous when estimated in the time-domain or frequency-domain, with the center frequencies mostly getting underestimated. Further, as expected, the errors in these estimates increase with bw and decreasing cf and decrease for smaller exponents (Fig. 1*b* vs Extended Data Fig. 1-1*b*).

A reported advantage of using FOOOF toolbox is to get unbiased estimates of various periodic components, including maximum power, cf, and bw. A suggested way to use FOOOF is to subtract the estimated aperiodic component and use the “flattened” spectrum to estimate the periodic components by fitting Gaussian peaks (Kala et al., 2023). Using FOOOF this way on the simulated data, the errors in cf estimates indeed went down to ∼3% in the theta frequency range (Fig. 1*c*), but the power and cf estimates were still visibly inaccurate (Extended Data Fig. 1-1*d*). Similar to previously used methods, errors in these estimates increase with bw, aperiodic exponent, and decreasing cf (Fig. 1*c*, Extended Data Fig. 1-1*c*).

To improve the periodic parameter estimates with FOOOF, we modified it to iteratively estimate the aperiodic components to obtain a flattened spectrum, fitting Gaussians to the periodic components, and subtracting the oscillatory parts from the original spectrum to estimate the aperiodic components again. The errors in estimates of the cf and the exponent of the aperiodic component decreased with the iterations (Fig. 1*d*), for the simulated theta peaks. After 20 iterations, the errors in the cf estimates dropped down to <0.1% (Fig. 1*d*,*e*; Extended Data Fig. 1-1*e*). To simulate peaks in the sgamma band, we put in a Gaussian peak with cf of 30–40 Hz and bw of 5–15 Hz. As expected, the iterative procedure does not affect the cf and exponent estimates of sgamma as much (Fig. 1*d*) and decreases the cf error estimates from 0.7 to 0.4% (Fig. 1*f*,*g*). Error estimates after iterations (Fig. 1*e*,*g*) remain close to 0, though their trends can vary visibly depending on the random seed used in the simulation.

### Improved aperiodic fitting until 200 Hz

Beyond estimating the periodic components, we further used FOOOF to analyze the aperiodic components of ephys signals from the mouse hippocampus. FOOOF allows doing this by fitting a Lorentzian function to the log of power in the signals varying with frequency. In its simplest form, this fitting would lead to linear 1/*f^n^* fitting of the signals in log(power)-log(frequency) scales (see Materials and Methods; Eq. 1), with the exponent *n* as a possible signature of the acquisition system or biologically relevant parameters. The exponent *n* has usually been estimated within a small range of frequencies, with recent reports arguing that *n* would depend on the frequency range of estimation (Boncompte et al., 2024). This exponent has been modeled in the range of 30–70 Hz, to be correlated with excitation to inhibition (E/I) ratio (Gao et al., 2017). We applied this linear fitting in log-log scales to LFP data attained using tetrodes in mouse hippocampus, specifically in CA1 and DG subregions (see Materials and Methods; purple and yellow boxes, respectively). Due to periodic components in hippocampal ephys signals going as high as 200 Hz (Buzsáki, 2015), we attempted to fit the aperiodic part to a large range of frequencies (4–200 Hz). This linear fitting (termed “1exp”) gave acceptable fits in smaller frequency ranges (4–100 Hz) but got qualitatively and quantitatively worse (see Materials and Methods, Errors estimation) when the frequency range was increased to 200 Hz (Fig. 2*a*, column 1). Further, on the same log-log axes, FOOOF also allows this fitting to have a “knee” frequency, with a flattened fit for lower frequencies, and a linear fit for higher frequencies (termed “flat + 1exp”; see Materials and Methods; Eq. 2). On using “flat + 1exp” fitting, we got more accurate fits for some datasets (Fig. 2*a*, column 2), with lower errors, but some datasets still had high errors (Fig. 2*a*, rows 2, 4, 5).

**Figure 3.**
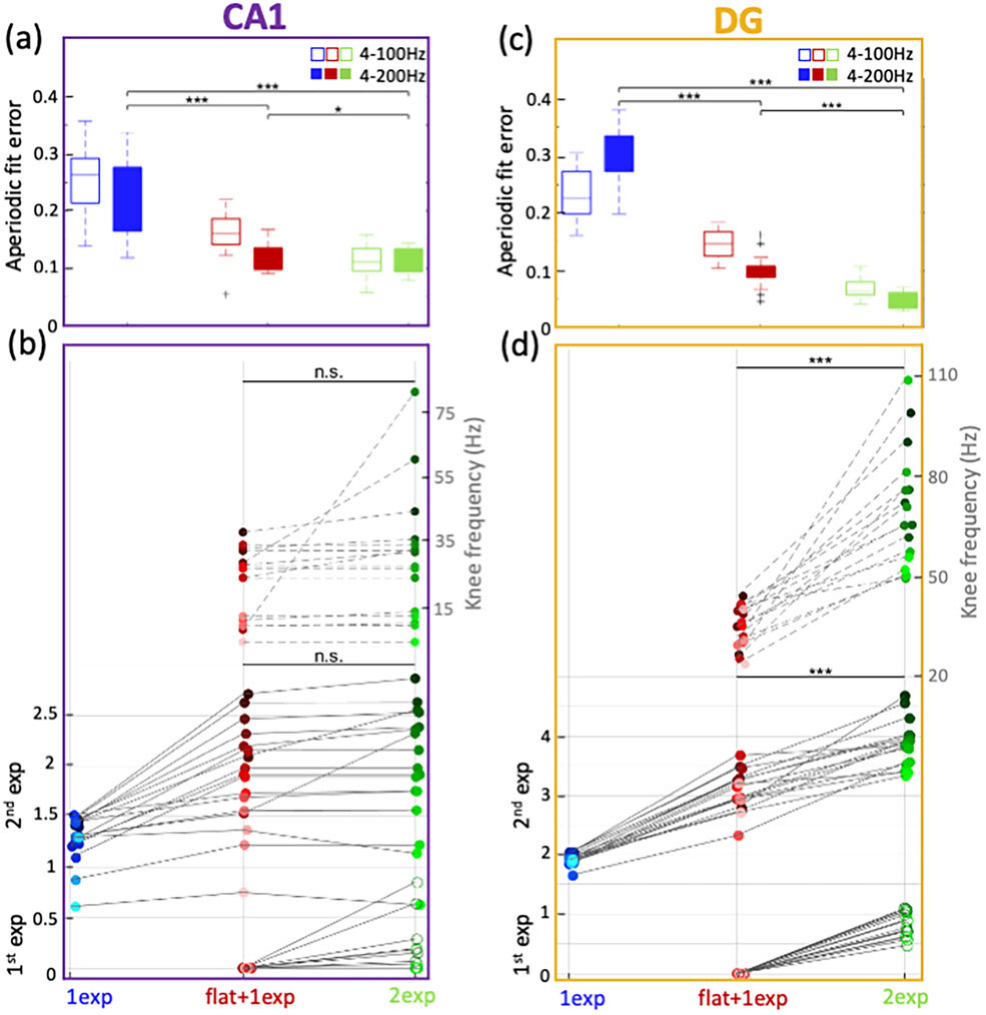
Comparison of aperiodic components between CA1 and DG. ***a***, Aperiodic fit errors for CA1 signals (*n* = 17) across “1exp”, “flat + 1exp” and “2exp”, when fit in the ranges of 4–100 Hz (open boxes) and 4–200 Hz (closed boxes). 4–200 Hz paired *t* test, *p*(“1exp”|“flat + 1exp”) = 1.50 × 10^−5^, *p*(“1exp”|“2exp”) = 4.20 × 10^−6^, *p*(“flat + 1exp”|“2exp”) = 0.0186. ***b***, Change in the aperiodic components for CA1 signals between the three fitting methods. A color gradient is added to visualize the mapping of each point across the fitting methods. The gradient is based on the order of points in the “2nd exp” population for “2exp” fitting. Paired *t* test, 2nd exponent: *p*(“flat + 1exp”|“2exp”) = 0.144, “knee” frequency: *p*(“flat + 1exp”|“2exp”) = 0.107. ***c***, Same as ***a*** but for DG signals (*n* = 17). 4–200 Hz paired *t* test, *p*(“1exp”|“flat + 1exp”) = 6.76 × 10^−12^, *p*(“1exp”|“2exp”) = 2.49 × 10^−13^, *p*(“flat + 1exp”|“2exp”) = 1.12 × 10^−6^. ***d***, Same as ***b*** but for DG signals. Paired *t* test, 2nd exponent: *p*(“flat + 1exp”|“2exp”) = 6.71 × 10^−7^, “knee” frequency: *p*(“flat + 1exp”|“2exp”) = 1.28 × 10^−6^. For all figures, *** *p* < 0.0005, ** *p* < 0.005, * *p* < 0.05, n.s., not significant. Further quantifications of our models can be found in Extended Data Figure 3-1.

As this method was originally optimized for EEG and ECoG signals, both of which are limited in sampling frequency (Gerster et al., 2022), restraining the maximum fitting frequency to 100 Hz would work well. To optimize this method to analyze ephys data and fit the signals to a large range of frequencies, we established new aperiodic fits. Observationally, ephys signals in 4–200 Hz were composed of two linearly varying bits with a varying “knee” frequency. We thus established a new fitting paradigm, termed “2-exponents” (“2exp”; see Materials and Methods; Eq. 3), providing the possibility for the data before the “knee” to have a nonzero exponent as well. Using this fitting function, we got better qualitative fits (Fig. 2*a*, column 3) and quantitatively lower errors. Applying “2exp” fits to hippocampal ephys signals slightly refined the fitting errors for CA1 (Fig. 3*a*, Extended Data Fig. 3-1*a*), but it substantially improved the aperiodic fit errors improved by ∼50% and full model fit errors by ∼25%, (Fig. 3*c*, Extended Data Fig. 3-1*b*) for DG signals, implying differential biological signatures between these regions. We found all aperiodic components from CA1 to be significantly lower than in the DG (Fig. 2*d*; mean CA1 parameters: 1st exp = 0.14, 2nd exp = 1.98, knee = 28.2 Hz; mean DG parameters: 1st exp = 0.84, 2nd exp = 3.88, knee = 69.6 Hz). The CA1 parameters were found to not be significantly different than in cortical ephys data (Fig. 2*b*–*d*; mean cortex parameters: 1st exp = 0.35, 2nd exp = 1.81, knee = 28.1 Hz). Some of these aperiodic parameters follow similar trends when using the “1exp” and “flat + 1exp” fits as well (Fig. 2*b*,*c*), but their exact values might be inaccurate due to worse fitting errors.

10.1523/ENEURO.0136-25.2026.f3-1 Figure 3-1 **Further quantification of our model fittings** (a)(top) Tetrode sites were confirmed post-mortem with histology (bottom). Full model fit errors for CA1 signals across ‘1exp’, ‘flat+1exp’ and ‘2exp’, when fit in the ranges of 4-100 Hz (open boxes) and 4-200 Hz (filled boxes). 4-200 Hz paired t-test, p(‘1exp’|’flat + 1exp’) = 5.33X10^−4^, p(‘1exp’|’2exp’) = 3.31X10^−4^, p(‘flat+1exp’|’2exp’) = 0.0547. (b) Same as (a) but for DG. (bottom) 4-200 Hz paired t-test, p(‘1exp’|’flat + 1exp’) = 2.97X10^−9^, p(‘1exp’|’2exp’) = 3.98X10^−11^, p(‘flat+1exp’|’2exp’) = 6.98X10^−6^. (c) Change in the aperiodic components for CA1-pyr and CA1-rad signals using ‘2exp’ fitting. Paired t-test, 2^nd^ exponent: p = 7.62X10^−4^, 1^st^ exponent: p = 0.0473, ‘knee’ frequency: p(‘flat+1exp’|’2exp’) = 0.135. (d) psds of 2 example DG signals, original and, after filtering and down-sampling. Dashed vertical lines at 200 Hz and 400 Hz. (e) The change in BIC scores across the 5 aperiodic fitting models, averaged over all CA1 and DG signals. For all figures, *** p < 0.0005, ** p < 0.005, * p < 0.05, n.s. not significant. Download Figure 3-1, TIF file.

Biological significance of these aperiodic parameters can be gleaned from previous literature. A recent study modeled the 1/*f^n^* exponent *n* between 30 and 70 Hz in hippocampal psd, to be correlated with the excitation–inhibition (E/I) balance (Gao et al., 2017). Using LFP data recorded in rats' CA1-stratum pyramidale (CA1-pyr) and CA1-radiatum (CA1-rad), and ECoG data from macaques, they showed that increasing E/I ratio flattened the exponent *n*. Although exponents from different models are not necessarily exchangeable or similarly interpretable, it is interesting to note that comparing LFP signals from mice's CA1 layers acquired with our setup (see methods) show consistent changes in the exponents (Extended Data Fig. 3-1*c*). Parallelly, as expected from literature (Arima-Yoshida et al., 2011; Alkadhi, 2019), with higher excitation and lower inhibition in CA1, the E/I ratio should be higher in CA1, potentially leading to flatter 1/*f* slopes in CA1 than in the DG, at least in the 30–70 Hz frequency range (see Discussion). On the other hand, the “knee” can be thought of as inversely related to different timescales in the data, which reflects the speed of synaptic and transmembrane currents (Gao et al., 2020; Zeraati et al., 2024). In humans and macaques, this is found to increase along the sensorimotor-to-association cortical axis (Gao et al., 2020). An increase in this timescale leads to a decrease in the “knee” frequency, leading to an inverse correlation in the “knee” frequency along this axis. Between the hippocampal subregions, higher propensity of complex burst spiking in CA1 than in DG should correlate with longer timescales and lower “knee” frequencies in CA1 than DG (Galashin et al., 2024). The “knee” frequencies found here in the mice hippocampal subregions translate to timescales of ∼7.2 ms in CA1 and 2.5 ms in DG, aligning with the known bursting characteristics of these regions (Harris et al., 2001). Thus, the aperiodic parameters we obtained within the hippocampal subregions are coherent with the differences between these subregions as reported in the literature.

### Aperiodic fitting beyond 200 Hz

**Figure 4.**
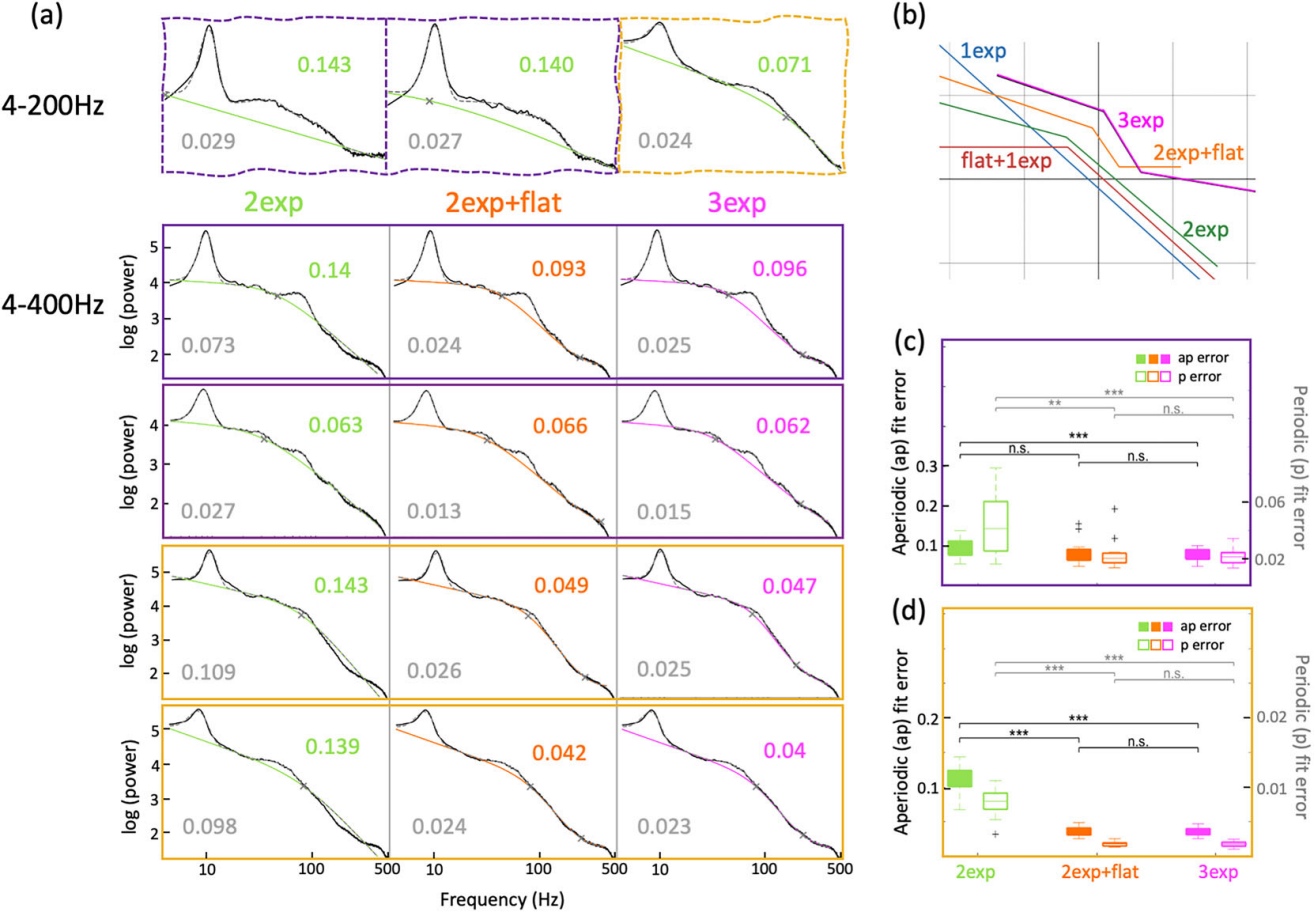
Aperiodic components in hippocampal electrophysiological signals between 4 and 400 Hz. ***a***, Row 1 shows “broken examples” of aperiodic fitting of mouse CA1 (purple) and DG (yellow) electrophysiological signals using “2exp” between 4–200 Hz. Rows 2–5 show similar examples until 500 Hz, fit using “2exp” (green), “2exp + flat” (orange), and “3exp” (magenta), between 4–400 Hz. Row 1, column 3 and Row 5 show the same dataset. ***b***, The general form of all five fitting functions. ***c***, Aperiodic (filled boxes, left *y*-axis) and full model (open boxes, right *y*-axis) fit errors for CA1 signals across “2exp,” “2exp + flat,” and “3exp,” when fit in 4–400 Hz. Paired *t* test, aperiodic errors: *p*(“2exp”|“2exp + flat”) = 0.122, *p*(“2exp”|“3exp”) = 5.81 × 10^−5^, *p*(“2exp + flat”|“3exp”) = 0.247; Full model errors: *p*(“2exp”|“2exp + flat”) = 1.82 × 10^−3^, *p*(“2exp”|“3exp”) = 2.94 × 10^−4^, *p*(“2exp + flat”|“3exp”) = 0.516. ***d***, Same as ***c*** but for DG signals. Paired *t* test, aperiodic errors: *p*(“2exp”|“2exp + flat”) = 1.24 × 10^−11^, *p*(“2exp”|“3exp”) = 1.44 × 10^−11^, *p*(“2exp + flat”|“3exp”) = 0.443; full model errors: *p*(“2exp”|“2exp + flat”) = 2.47 × 10^−10^, *p*(“2exp”|“3exp”) = 2.80 × 10^−10^, *p*(“2exp + flat”|“3exp”) = 0.476.

Periodic components in ephys recordings might extend beyond 200 Hz, such as sharp-wave ripples in CA1 and CA3 (Buzsáki, 2015). Due to this, some example signals had qualitatively worse fits and higher fitting errors until 200 Hz (“broken examples”; Fig. 4*a*, top row). Thus, to obtain complete fits of the periodic components, we further fit our data until 400 Hz (see Materials and Methods; Extended Data Fig. 3-1*d*). Applying “2-exp” aperiodic fitting to hippocampal mouse data until 400 Hz, gave much poorer fits with higher errors (Fig. 4*a*,*c*,*d*). To improve this, we further developed two more aperiodic fits to cover the whole range of frequencies from 4 to 400 Hz. To match the observational aperiodic nature of the data extending into these higher frequencies, we added a third linear piece to the above curves, adding a second “knee” and potentially a third exponent. For the first of these fits, we fixed the third exponent to be 0, i.e., have a flat end to the spectrum, thus naming this fit as “2exp + flat.” We also used another fit, with the third exponent also be a free fitting parameter, thus naming this “3exp” (see Materials and Methods; equations 4,5).

Fitting until higher frequencies helped with better estimates of the periodic components, such as higher power in specific oscillations or detectability of very low power oscillations (Fig. 4*a*). Examples that could not be fit well with previous models (“broken examples”; Fig. 4*a*, top row, column 3), fit much better at higher frequencies with the two new models (Fig. 4*a*, fourth row), and errors got better with both these models (Fig. 4*c*,*d*). The general form of all the fitting functions is shown in Figure 4*b*. Though for majority of the cases, both “2exp + flat” and “3exp” functions behave similarly, some cases preferentially fit better with one or the other (Fig. 4*a*, third row), providing flexibility in fitting a wide variety of signals without prior assumptions.

To compare the efficacy of the fitting models, we compared the BIC scores (Extended Data Fig. 3-1*e*; see Materials and Methods; Eq. 6) for the first three models between 4 and 200 Hz and for the last two models between 4 and 400 Hz. The change in BIC scores clearly show that the more complex models fit the data better, with the increasing complexity being particularly critical for the analyses of DG signals.

## Discussion

With crucial modifications to the open-source toolbox FOOOF, we decreased the estimation errors for periodic components while also assessing the aperiodic components of mouse hippocampal electrophysiological signals, not only for a narrow range of frequencies, but also within a wide range from 4 to 400 Hz.

For the assessment of the periodic components, using the conventional methods that do not estimate the aperiodic fits of the neural data, such as bandpass filtering or Chronux toolbox, the estimates of the cf of the brain oscillations will always be underestimated (Fig. 1*b*). This underestimation would further increase for oscillations with lower frequencies and at higher aperiodic exponents. Correctly estimating these frequencies is essential to compare conditions that cause only small changes in the brain rhythms.

We demonstrated that FOOOF tends to slightly overestimate high-power peaks in the low-frequency range (Fig. 1*c*), which is a natural consequence of the initial aperiodic fit being partially affected by the periodic component. Iteratively reducing the impact of the respective other component during periodic and aperiodic fitting visibly improved estimation of these low-frequency periodic components (Fig. 1*d*–*e*). The improvement of this approach was particularly evident at theta frequencies and progressively diminished in the slow gamma range. As limitation of our study, it is unclear how robust our method is with regard to model violations (i.e., asymmetrical oscillatory peaks or inappropriate aperiodic models) and/or different levels and nature of noise.

Even though the improvement in errors with our iterative FOOOF procedure was seemingly small (3–5% in the theta range), it is comparable to the changes in theta cf associated with behavioral parameters, such as speed and acceleration (Kennedy et al., 2022). As the frequencies of these rhythms are also thought to be correlated with the speed of interregional synchrony (Ahmed and Mehta, 2012), small changes in these might have important biological significance. Our methods would thus be crucial in accurately correlating changes in brain rhythms with animal's cognitive states or behavioral contingencies.

Our improved toolbox was critical for a complete comparison of the aperiodic parameters between different hippocampal subregions. At low frequencies, the exponents were found to be small (<1), but nonzero for a few CA1 signals (8/17), while they were always nonzero for all DG signals (1st exp comparisons; Fig. 3*b*,*d*). While the 1/*f* trend in a certain frequency range is often approximated by either a fixed exponent (“1exp”) or a single Lorentzian (“flat + 1exp”) fit (Donoghue et al., 2020b; Gao et al., 2020), recent foundational modeling has suggested that broadband aperiodic spectra can arise from the superposition of multiple exponentially decaying processes, which in the frequency domain can be approximated by a sum of Lorentzian functions. For instance, Gao et al. (2017) focused on the different decay constants of GABA and AMPA currents, resulting in two overlapping Lorentzian functions with different knee frequencies, concluding that their relative quantity will be reflected in their offset and hence the linear slope in the overlapping frequency range. Chaudhuri et al. (2018), on the other hand, demonstrated how recurrent networks tend to produce both short timescales, representing fast-decaying activity of local neuronal populations, and long timescales, representing the maintenance of global network states arising from recurrent connectivity near criticality. A double-Lorentzian model has also been adapted by Brake et al. (2024), who demonstrated how both modulatory changes of synaptic time constants and network connectivity can influence the aperiodic component.

Taking these findings together and considering that both synaptic decay constants (Destexhe et al., 2003) and network-level activity decay (Gao et al., 2020) constants span broad ranges, it becomes evident that the biological aperiodic spectrum is influenced by a larger range of slow timescales. This range of timescales might converge into an aperiodic spectrum that can be well approximated by the double-exponential model we suggested and which has previously been reported to well match the shape of MEG spectra across the cortex (Chaoul and Siegel, 2021). This consideration would support the idea of the first exponent being influenced by a range of slower timescales and the knee frequency marking the transition into a dominant fast timescale, yet the underlying physiological processes that might be reflected by these measurements are unclear. While differences in E/I balance have been proposed to influence spectral slopes in a restricted frequency range of 30–70 Hz (Gao et al., 2017), this interpretation seems limited given the broader frequency range and the deliberate flexibility used for our modeling approach. One plausible contributor to longer effective timescales in CA1 is its higher degree of recurrent connectivity, which has been shown to give rise to slower, more persistent network dynamics (Chaudhuri et al., 2018). In addition, anatomical differences, differential dendritic integration properties (Lindén et al., 2010; Pettersen et al., 2014), and stochastic fluctuations in ion-channel activity (Diba et al., 2004) may further contribute to shaping the aperiodic spectrum across different frequency regimes. A complete picture of the biological implications of the differences between aperiodic parameters of CA1 and DG will require further investigation.

For most of the signals from CA1, the aperiodic parameters did not change at all or by only a little between the “flat + 1exp” and “2exp” methods (Fig. 2*c*). Conversely, they changed significantly for the DG signals (Fig. 3*b*). This shows that while the aperiodic fitting as done within the original FOOOF toolbox might work for EEG and hippocampus CA1 ephys data, our modifications are essential for DG ephys data. This is also evident in the change in BIC scores (Extended Data Fig. 3-1*c*).

The use of alternative approaches like IRASA to isolate aperiodic components (Wen and Liu, 2016), in combination with aperiodic modeling as demonstrated by Chaoul and Siegel (2021), was not investigated in our study. In addition, our findings were not tested by cross- or split-sample validation by SPRiNT for spectral parameterization (Wilson et al., 2022). Addressing these limitations will be important in future studies for benchmarking our modeling approach for hippocampal aperiodic components. Provided additionally that our mathematical models are confirmed in future studies with the inclusion of higher number of animals, recording sessions, and behavioral data, our study can be instrumental for the better assessment of brain activity and function and valuable to assess changes in periodic and aperiodic parameters, such as center frequency, exponents, and “knee” frequencies, to various behavioral and cognitive states, such as during hippocampal memory tasks, sleep replay, aging, performance in virtual reality (Purandare et al., 2022), etc. Further, recent reports suggest that the E/I ratio might not be uniform throughout the DG, with its upper blade having higher inhibition than its lower blade (Luna et al., 2019; Berdugo-Vega et al., 2023). Our analyses methods as outlined in this work may be valuable in the in investigation of blade-specific differences in aperiodic parameters, capturing potential contributions from both E/I balance and differences in effective neuronal and network timescales.

## Synthesis

Reviewing Editor: Silvia Pagliardini, University of Alberta Decisions are customarily a result of the Reviewing Editor and the peer reviewers coming together and discussing their recommendations until a consensus is reached. When revisions are invited, a fact-based synthesis statement explaining their decision and outlining what is needed to prepare a revision will be listed below. The following reviewer(s) agreed to reveal their identity: Sachin Deshmukh, Thomas Donoghue. Note: If this manuscript was transferred from JNeurosci and a decision was made to accept the manuscript without peer review, a synthesis may not be available.

The manuscript provides a substantial improvement over the FOOF method used for segregating periodic and aperiodic components from the LFPs, but the authors need to provide evidence that this improvement aids in answering real biological questions.

Reviewer#1

Kuhn et al. describe an enhancement of FOOF protocol to segregate aperiodic and periodic signals in the frequency domain. They use an iterative procedure to fit a variety of models to aperiodic data - single exponent, flat + exponent, two exponents, two exponents + flat, and 3 exponents and show that two exponents + flat, and 3 exponents produce fits with least errors in periodic as well as aperiodic signal estimation.

While I have a number of minor methodological concerns, my major concern is about the significance and interpretation of the data.

Major concerns:

1. The authors cite Gao et al. 2017 (P10) to imply that the differences between the exponents in CA1 and DG arise out of differences between EI balance between the two. This is a huge leap from what Gao et al. 2017 showed. Gao et al 2017 demonstrated how change in E/I balance changed the exponent of 1/f using simulations. Since they changed E/I balance for the same region, other variables such as filtering properties of the region, orientations of the dipoles and other neuroanatomical features etc. remain unchanged. The present article is doing the comparison across two different regions and, as such, must account for these other differences as well, while explaining the difference in exponent. In fact, the authors talk about the differences in synaptic transmembrane current speed to account for the differences in knee parameter in this paragraph itself. Do these not affect the exponent of 1/f?

Gao et al. 2017 deal with many of these issues in their discussion of limitations of their model, in the context of changing EI balance within the CA1 network. How do these limitations affect when comparing networks anatomically and physiologically and as dissimilar as DG and CA1?

2. The authors show a small but statistically significant improvement in the accuracy of segregation with their method. However, they fail to empirically substantiate their claim that the improved accuracy of estimation of aperiodic as well as periodic signals would help differentiate small but biologically relevant differences in signals. Demonstrating a comparison in which FOOF fails to discriminate between LFPs recorded from the same region under two experimental conditions while the author's modification of the procedure enables this distinction would convince the readers far more than the statistically significant but small differences in estimates.

Minor concerns:

1. Electrophysiology and Neural Signal Acquisition section in the methods (P3) needs to specify the bandpass filter range used.

2. The authors downsample LFP traces at 1KhZ (P3). Do they filter the data to prevent aliasing before downsampling? Depending on the higher cutoff of the bandpass filter used while acquiring data, this step may critically alter the data.

3. P3: "Electronic noise peak at 50Hz was removed from the psd by applying the '1exp' FOOOF fit in a small range (43-57Hz) and subtracting the fitted Gaussian." How strong was the line noise to necessitate this? Need confirmation that this process, including removal of harmonics isn't introducing its own artifacts.

4. It is not clear if the description in the simulated data section on P3 is talking about simulating a time domain aperiodic as well as periodic signal, or if only the aperiodic signal is generated as a function of time and gaussian peaks at theta/gamma/line noise are added to the PSD of the aperiodic signal.

5. In the same section, the authors state that the aperiodic signal was modelled as a 1/f^n random noise in the 1-1000 Hz range. Was the experimental data not filtered to be within the nyquist limit for the 1000hz downsampling mentioned on P3? If it was, isn't the simulation overestimating ~500 (or whatever the low pass filter they used before downsampling)-1000Hz amplitude in the real data?

6. P4: "Simplified versions of the equations are shown here for clarity". Exact equations must be shown somewhere in the paper or the supplement. The authors may choose to show the simplified versions in addition, but not instead of, the actual equations used.

7. P4: "For direct comparison of the '2exp' fitting with 'flat+1exp' fitting, we put the following conditions on the aperiodic components after the fittings: if the absolute difference between the two exponents is less than 0.01, or if the reported 'knee' frequency is greater than 195Hz, we change the 1st exponent to be 0, 2nd exponent to be the as-obtained value and the knee frequency to 4Hz." This procedure raises a possibility that this subjective tweaking of the model itself might be adding differences in features detected. The authors need to control for this possibility.

8. P5: "To avoid over-fitting until 500Hz, we implemented step-wise curve-fitting, i.e. fit the data until 200Hz with the '2exp' function, fixed the '1st exponent', '1st knee frequency' and starting power at 4Hz, which might be biologically more relevant, and then re-fit the data. This allows the 2nd and 3rd exponent, and the 2nd knee, to be fit freely. This step-wise fitting can prevent mathematical operations from messing up biologically relevant parameters." This claim needs to be empirically verified using modelled data.

9. P5: "We started both aperiodic and periodic assessment at 4Hz to avoid delta rhythm, which can be inconsistent in the hippocampus." citation or empirical evidence is needed for this claim. Why are the authors eliminating frequencies where there is a lot of power but going to extra lengths to estimate higher frequencies (>250Hz) where there's very little power and high likelihood of contamination by action potentials in hippocampal LFPs?

10. P5: "instead of using FOOOF's multi-Gaussian fitting method, we divided the flattened spectrum into hippocampus relevant oscillatory bands and ran the peak detection process only once per band. This was done to avoid an individual brain rhythm being represented as a combination of multiple Gaussians, which posed a challenge for further analyses and interpretation." Empirical evidence is needed to show that peak is a better indicator of amplitude in an oscillatory band than power in the whole band - gaussian fitted or otherwise. Also, examples where FOOF returned multiple gaussians where there was only a single oscillation in the flattened spectrum need to be shown along with comparison of the peak amplitudes obtained using the authors' method with the standard FOOF method.

11. P6: "The parameters for simulations were chosen based on the medians in real tetrode ephys data from the mouse hippocampus." PSDs of experimental data with fits need to be shown in fig 1 for comparison with the simulated data.

12. Fig 1a: 2hz and 5hz bw labels for 6hz cf are reversed. Is the aperiodic signal used identical for all 4 examples shown? Is that the case with all the simulations? If so, do the results hold when aperiodic signal is randomised?

13. Fig 1c: What was randomised and how? Unclear from the legend.

14. Aperiodic fitting until 500 Hz (P10): These higher frequencies have substantial contributions from action potentials. How do the authors account for those when using the aperiodic fits to estimate EI balance etc. from LFPs?

15. Fig 3 a,b need to be right next to fig 2 b,c to facilitate direct visual comparison between CA1 and DG. This can be achieved by merging figs 2 and 3.

16. Tick/axis labels and keys within many figures have very small fonts. These should be increased to match those used in Fig 2a.

Reviewer #2

In this manuscript, Kühn and colleagues investigate the application of the fooof / spectral parameterization algorithm to rodent hippocampus data. In doing so, they explore a series of changes / additions to the algorithm, including adding additional iterations and bounds for estimate the periodic components, and adding additional model forms for estimating the aperiodic component. With this updated approach, they investigate data recorded from rodent hippocampus, showing that these updated approaches assist with better fitting such data. They compare the empirical fits across the examined areas and note some differences, in particular in the aperiodic components, between areas CA1 and DG. Overall, I find this manuscript addresses an important question of how best to characterize LFP data as recorded from animal models and provides some useful updates and additions to methods for doing so while motivating these changes with empirical examples. In the current version, I do find there are currently some limitations to some of the descriptions in the manuscript that need addressing before this paper is ready for publication.

Comments

There are some limitations to the methods reporting, including that Figure 3 shows power spectra from cortex, but this is not otherwise described in the paper. If cortex recordings are included, this should be described in the methods. Where in the cortex are these recordings from? Are cortex recordings from the same tetrodes before they were lowered into the hippocampus, or some other set of electrodes?

It is not clear how the component specific errors are computed. The methods report that {aperiodic, periodic} error is computed as the mean absolute difference between "data and the {aperiodic / periodic} fits". Is this with the full data? This doesn't make sense - the error between the aperiodic-only model fit component and the full data would be dominated by the peaks and (due to different peaks across different spectra) this is not comparable across spectra. Perhaps the computation is between the model component and the isolated data components (e.g. ap_data = full spectrum - pe_model). If so, this needs to be clarified, and further discussed, including a note of the properties and limitations of such an approach - perhaps most notably that (if it is indeed using the isolated data components), such a measure is not actually necessarily specific to the component under study in that the 'ap_data' may be dissimilar from the 'ap_model' either because the 'ap_model' is a bad description of the aperiodic component specifically and/or because the 'pe_model' is a bad description of the periodic component, and due to this 'ap_data' is not actually a good estimate of the aperiodic component, regardless of 'ap_model'. Note that the original spectral parameterization method does not describe or use any such component specific metrics (likely due to these issues) and so if developing and applying new metrics here, the paper needs to describe, motivate, and validate them.

The paper uses some simulations to evaluate the changes to the fitting method, which is an important way to demonstrated and validate the methodological changes. However, the simulations appear very limited - it would appear the simulations only test high powered peaks with a specific exponent and no noise? If so, such a limited range of simulations is not adequate to make the general claim, as is stated, that these modifications improve periodic component fitting in the way they do, without noting the specificity of the simulations and test cases examined. If the claim is that the change in methods is general, a much broader range of simulations is required, as it is not clear that the finding in this specific simulation regime generalizes (note that the original fooof method is targeted at addressing a broad range of potential spectra, as exemplified by the broad range of simulations explored in the paper). In the context of this paper, it would also seem appropriate to note that the goal is specifically testing a specific context of the pattern seem in the empirical data under study, but then the claims made about the performance need to be appropriately discussed in terms of the scope of this demonstration, and the generalizability (or potentially, lack thereof) should be noted.

In addition, there are some aspects of the figure presentation that are a bit unclear and could perhaps be ameliorated:

- In Figure 1 it would be useful to put titles on the panels to clarify what is being plotted

- The organization of results across figures is a little unclear. Fig 2BC and Figure 3AB are (I think?) the same analysis for CA1 and DG respectively? It's unclear why these are split across different figures. This also seems inconsistent with the figure titles - Figure 3 is described as a comparison between CA1 &DG, but 3AB is not a comparison between regions.

- Figure S1 incorrectly uses a sequential color map for data that crosses 0 (A, B) and should therefore us a diverging colormap

The paper notes the previous literature linking measures of the aperiodic exponent to the E/I relationship, in particular from the Gao, 2017 paper. At times, notably at the end of the discussion, it seems to imply that this interpretation from the Lorentzian model of that paper can be applied to the results in the current paper, which use different model forms. It is not one clear can take the findings of Gao, 2017 with one particular model, and apply it to a different model form, as the measured parameters are not interchangeable and the way they relate to underlying physiology may differ. For example, how does one map the exponent of the Lorentzian knee model to the exponents of, for example, the 3exp model in DG? If the E/I link is to be evoked for the new empirical results here, if and how this interpretation relates to the prior work requires at least some additional discussion.

The introduction claims there are only two reports of rodent hippocampus - how was this determined? This doesn't seem to include for that the Gao, 2017 paper examined rodent CA1 (this is noted later in the paper), and seems likely to be an undercount.

Methods: How much data was analyzed? The number of epochs / length of time of data that was included need to be reported in the methods.

Across the manuscript, there are some phrasings or descriptions relating to spectral parameterization that I find hard to understand in terms of what they specifically are meant to refer to, and in some cases seem may misreport details of the previous methods which require clarification and/or correction:

- Introduction: What does aperiodic parameters are 'multi-fold' mean?

- Methods / Simulated data section: what does "heighten the fallacies of FOOOF" mean / refer to?

- Methods / Detection of periodic components: "This modification is different from the 'Band-by-Band' approach introduced with FOOOF 2.0, namely specparam, which relies on removing unwanted Gaussian peaks in post-analysis." It is unclear what this refers to?

- The manuscript describes the fooof single exponent model as "linear fitting". This is incorrect - while the model is equivalent to the linear fit of log-log power spectrum, this is not what the fitting process is, which fits 1/f functions in semi-log space.

## Author Response

We are thankful to the reviewers for their valuable feedback. All their comments and concerns were very helpful in improving the quality of this work. Accordingly, we have made modifications to the manuscript (highlighted text), and have additionally replied to some of their comments below (in blue, location of modification in manuscript indicated in bold).

Synthesis Statement for Author (Required):

The manuscript provides a substantial improvement over the FOOF method used for segregating periodic and aperiodic components from the LFPs, but the authors need to provide evidence that this improvement aids in answering real biological questions. **Our work pivots on providing a mathematically rigorous method of assessing the aperiodic parameters of hippocampal LFP over a wide range of frequencies. The previous methods (such as FOOOF), even if applied to this large range of frequencies, would give estimates with higher errors, as shown with our aperiodic and periodic errors.

To the best of our knowledge, complete estimation of the aperiodic components in the rodent hippocampus has never been reported before. Though these components have shown to be correlated with task demands, age and disease, but again, never clearly for rodent hippocampal signals. The psd exponents estimated with our improvements in the rodent hippocampal sub-regions, could potentially be correlated with their known excitation-inhibition ratios (Arima-Yoshida et al., 2011; Alkadhi, 2019), which is the only known correlate of these exponents in the rodent literature (Gao et al., 2017). We have now better highlighted these claims in the results and discussion sections (P8, 10).

Further, the changes in both periodic and aperiodic parameters with experimental paradigms can be very subtle, with high variance. For instance, in the manuscript, we only include data with animal's movement speeds above 5cm/s (stated in methods), as is usually done for hippocampal ephys data, to avoid confounding variation in hippocampal theta and possibly the aperiodic parameters at lower speeds. To exemplify, below we show LFP from two tetrodes in the DG, while the animal's moving speeds are within 1-4cm/s. In the LFPs below, FOOOF's fittings either would not have detected a theta peak (in blue using '1exp'), or would have estimated an erroneous peak (in red using '1exp+flat'). On the other hand, our improved models reliably detect a small-power theta peak. We have not included these figures and such claims in the manuscript, as we do not have a "ground truth" of the power or center frequency of theta in the DG, which is only characterized in CA1 (Kennedy et al., 2022) yet. Thus, to assess the importance of our fitting models, future studies with experimental manipulations will be needed to fully understand the biological significance of these parameters.

In summary, though we are aware that our work only provides limited evidence of the biological relevance of this improved method, we believe our models provide robust results that could foster many future experimental investigations.

Reviewer#1 Kuhn et al. describe an enhancement of FOOF protocol to segregate aperiodic and periodic signals in the frequency domain. They use an iterative procedure to fit a variety of models to aperiodic data - single exponent, flat + exponent, two exponents, two exponents + flat, and 3 exponents and show that two exponents + flat, and 3 exponents produce fits with least errors in periodic as well as aperiodic signal estimation.

While I have a number of minor methodological concerns, my major concern is about the significance and interpretation of the data. **Please see general comments above.

Major concerns:

1. The authors cite Gao et al. 2017 (P10) to imply that the differences between the exponents in CA1 and DG arise out of differences between EI balance between the two. This is a huge leap from what Gao et al. 2017 showed. Gao et al 2017 demonstrated how change in E/I balance changed the exponent of 1/f using simulations. Since they changed E/I balance for the same region, other variables such as filtering properties of the region, orientations of the dipoles and other neuroanatomical features etc. remain unchanged. The present article is doing the comparison across two different regions and, as such, must account for these other differences as well, while explaining the difference in exponent. In fact, the authors talk about the differences in synaptic transmembrane current speed to account for the differences in knee parameter in this paragraph itself. Do these not affect the exponent of 1/f? Gao et al. 2017 deal with many of these issues in their discussion of limitations of their model, in the context of changing EI balance within the CA1 network. How do these limitations affect when comparing networks anatomically and physiologically and as dissimilar as DG and CA1? **We have now modified the results and discussion to highlight these limitations and better link our work with previous studies (P10). First, we added new data and a corresponding supplementary figure (Figure 3-1c) to replicate the experimental claims from Gao et al., 2017, wherein they compare the exponents of psd from CA1-pyramidale and CA1-radiatum to their known synaptic densities. Using FOOOF, they were only able to compare and correlate these parameters for a very limited frequency range (30-70Hz); while using our improved fittings, we compared these across the whole range of 4-200Hz. Second, while making a complete estimation of all aperiodic components, we confoundingly see clear 'knee' frequencies in the range of 30-70Hz, as was also seen in Gao et al., but left unaddressed. Third, we have now added that anatomical differences and differential dendritic integration processes (Lindén et al., 2010; Pettersen et al., 2014), recurrent network activity (Chaudhuri et al., 2018) and stochastic fluctuations in ion channels (Diba et al., 2004) in these sub-regions might also contribute to the psd slope.

2. The authors show a small but statistically significant improvement in the accuracy of segregation with their method. However, they fail to empirically substantiate their claim that the improved accuracy of estimation of aperiodic as well as periodic signals would help differentiate small but biologically relevant differences in signals. Demonstrating a comparison in which FOOF fails to discriminate between LFPs recorded from the same region under two experimental conditions while the author's modification of the procedure enables this distinction would convince the readers far more than the statistically significant but small differences in estimates. **Please also see general comments above.

In a small range of frequencies, FOOOF's fitting will lead to the same results as with our models. This was indeed found to be true when we tried our fittings with EEG data (not shown in the manuscript).

For the periodic components, we clearly show in Figure 1 that our iterative estimation leads to lower errors with simulated data (where we know the "ground truth").

For the aperiodic components, we assert that our modifications enable us to find these components in wider frequency ranges that the original FOOOF would have been incapable of doing, or at least would have led to erroneous estimates.

Minor concerns:

1. Electrophysiology and Neural Signal Acquisition section in the methods (P3) needs to specify the bandpass filter range used. **Added the details in Materials and Methods (Preprocessing of neural data, P3,4) now.

2. The authors downsample LFP traces at 1KhZ (P3). Do they filter the data to prevent aliasing before downsampling? Depending on the higher cutoff of the bandpass filter used while acquiring data, this step may critically alter the data. **We have now put in a 500Hz low-pass filter. This did not change our results significantly, but we have updated our methods (P3,4) and results accordingly, maximally fitting only until 400Hz. We also added a corresponding Figure 3-1d to compare the performance of this filter with un-filtered non-downsampled original data (ground truth).

3. P3: "Electronic noise peak at 50Hz was removed from the psd by applying the '1exp' FOOOF fit in a small range (43-57Hz) and subtracting the fitted Gaussian." How strong was the line noise to necessitate this? Need confirmation that this process, including removal of harmonics isn't introducing its own artifacts. **As mentioned in methods and the results, the simulated spectra in Figure 1a closely represents the real data, which shows the 50Hz noise to be about 2.5dB. This is now also clear in the real data examples in the same figure. Further, estimation of simulated parameters was done after the removal of these peaks, resulting in the reported low errors - also showing that the removal of noise peaks does not introduce new artifacts. We have now also added Figure 1-1f showing that the aperiodic parameters do not differ significantly, with or without removing these peaks.

The removal of noise peaks by subtracting fitted Gaussians is implicitly done in FOOOF as well. As our detection of the periodic components allows fitting only one peak in separate bands (as mentioned in methods and Table 1), of which 48-52Hz was deliberately avoided, we did this removal step explicitly during pre-processing.

4. It is not clear if the description in the simulated data section on P3 is talking about simulating a time domain aperiodic as well as periodic signal, or if only the aperiodic signal is generated as a function of time and gaussian peaks at theta/gamma/line noise are added to the PSD of the aperiodic signal.

5. In the same section, the authors state that the aperiodic signal was modelled as a 1/f^n random noise in the 1-1000 Hz range. Was the experimental data not filtered to be within the nyquist limit for the 1000hz downsampling mentioned on P3? If it was, isn't the simulation overestimating ~500 (or whatever the low pass filter they used before downsampling)-1000Hz amplitude in the real data? **Clarified both points in Materials and Methods (Simulated data, P4) now.

6. P4: "Simplified versions of the equations are shown here for clarity". Exact equations must be shown somewhere in the paper or the supplement. The authors may choose to show the simplified versions in addition, but not instead of, the actual equations used. **The journal guidelines state "Only extended data directly related to figures or tables and corresponding figure legends are allowed". We have thus put the exact equations in the open-source code on Github (P6, mentioned in Code accessibility: The code/software described in the paper is freely available online at [https://github.com/huRashidy/LFP_FOOOF/tree/main]).

Further, the computational versions used do not convey the mathematical form of these equations, though being completely equivalent. For instance, for the '2exp' function, the equation used for fitting parameters was ys = knee_off - np.log10(10**(exp1*(np.log10(f)-np.log10(k))) + 10**(exp2*(np.log10(f)-np.log10(k)))) which when simplified using mathematical properties, such as log(AB) = log(A) + log(B), lead to the same form as in equation (3) in the manuscript. Thus, we have refrained from providing the exact equations in the manuscript as seeing the mathematical form is much more informative than the computational form. On the Github page, we have also provided any parameter redefinitions used to obtain the computational forms of these equations.

7. P4: "For direct comparison of the '2exp' fitting with 'flat+1exp' fitting, we put the following conditions on the aperiodic components after the fittings: if the absolute difference between the two exponents is less than 0.01, or if the reported 'knee' frequency is greater than 195Hz, we change the 1st exponent to be 0, 2nd exponent to be the as-obtained value and the knee frequency to 4Hz." This procedure raises a possibility that this subjective tweaking of the model itself might be adding differences in features detected. The authors need to control for this possibility. **This was done only for the purpose of plotting, and not during the model fitting of the signals, thus there is no 'subjective tweaking'. We have now added that clarification to Materials and Methods (Equations for the aperiodic fits, P5).

8. P5: "To avoid over-fitting until 500Hz, we implemented step-wise curve-fitting, i.e. fit the data until 200Hz with the '2exp' function, fixed the '1st exponent', '1st knee frequency' and starting power at 4Hz, which might be biologically more relevant, and then re-fit the data. This allows the 2nd and 3rd exponent, and the 2nd knee, to be fit freely. This step-wise fitting can prevent mathematical operations from messing up biologically relevant parameters." This claim needs to be empirically verified using modelled data. **We are not making a new claim here. Over-fitting with models with 5/6 free parameters is a common occurrence. We believe that the aperiodic parameters found until 200Hz, and their differences between CA1 and DG, are biologically significant as correlates to excitation-inhibition ratios and speeds of synaptic and transmembrane currents. Thus, to prevent over-fitting with the more complicated models, we restrain the parameters which we believe to be more important for a biological understanding. We have clarified the original statement to eliminate any confusion (P5).

9. P5: "We started both aperiodic and periodic assessment at 4Hz to avoid delta rhythm, which can be inconsistent in the hippocampus." citation or empirical evidence is needed for this claim. Why are the authors eliminating frequencies where there is a lot of power but going to extra lengths to estimate higher frequencies (>250Hz) where there's very little power and high likelihood of contamination by action potentials in hippocampal LFPs? **Hippocampal tetrodes do not have a lot of delta power during active exploration, especially as we are only taking epochs where the animal's speeds were >5cm/s. Added references for this in the same paragraph (P5). This can now also be seen in the 'real examples' in Figure 1a. We have now modified our fittings to only go until 400Hz, and usually electrophysiological data are filtered above that for action potential contributions. As stated, and can be seen in Figure 4, our purpose to fit above 200Hz is to allow any further adjustment of the aperiodic parameters found until 200Hz (such as 2nd exponent), which might slightly change when fit until higher frequencies. Thus, for completion, it is best to fit the whole range.

10. P5: "instead of using FOOOF's multi-Gaussian fitting method, we divided the flattened spectrum into hippocampus relevant oscillatory bands and ran the peak detection process only once per band. This was done to avoid an individual brain rhythm being represented as a combination of multiple Gaussians, which posed a challenge for further analyses and interpretation." Empirical evidence is needed to show that peak is a better indicator of amplitude in an oscillatory band than power in the whole band - gaussian fitted or otherwise. Also, examples where FOOF returned multiple gaussians where there was only a single oscillation in the flattened spectrum need to be shown along with comparison of the peak amplitudes obtained using the authors' method with the standard FOOF method. **We have modified the original sentence pointed here for clarity (P5).

Whether "peak is a better indicator of amplitude in an oscillatory band than power in the whole band" is an open question in the field of electrophysiology (Myrov et al., 2024) and is beyond the scope of the work presented here.

As we do not have the ground-truth in real data, we do not make any claims about if the peaks should be single or multiple Gaussians, or their estimated peak amplitudes. Please find an example below of fooof multi-Gaussian fitting and our single Gaussian fitting on the same flattened spectrum. As mentioned, single peak detection in each band was done for ease of further analyses and interpretation.

Lastly, our improvements with iterative peak detection (as shown in Figure 1), and better suited mathematical models for aperiodic parameters (as shown in Figure 2-4) are valid even with the standard FOOOF peak detection. Thus, we have not added the plots above to the manuscript, as they digress from the main points of the work presented here.

11. P6: "The parameters for simulations were chosen based on the medians in real tetrode ephys data from the mouse hippocampus." PSDs of experimental data with fits need to be shown in fig 1 for comparison with the simulated data. **Added psds of 3 real datasets in Figure 1a. Fits of multiple such datasets are shown in Figure 2 and 4 anyway.

12. Fig 1a: 2hz and 5hz bw labels for 6hz cf are reversed. Is the aperiodic signal used identical for all 4 examples shown? Is that the case with all the simulations? If so, do the results hold when aperiodic signal is randomised? **Thanks for correcting - fixed the 2Hz, 5Hz labels.

- For Figure 1, the aperiodic signal is kept the same as pink noise with exponent 1.2, as mentioned in the caption and the methods.

- As shown in Figure 1-1c, the errors increase with an increase in exponent, as expected. Explicitly stated this in Results (P7) now.

13. Fig 1c: What was randomised and how? Unclear from the legend. **Clarified now (Figure 1 legend) - we averaged over 20 initiations of simulated data.

14. Aperiodic fitting until 500 Hz (P10): These higher frequencies have substantial contributions from action potentials. How do the authors account for those when using the aperiodic fits to estimate EI balance etc. from LFPs? **Please see replies to points 8 and 9 above. We have now also clarified in the Discussion that the aperiodic behavior at high frequencies might be correlated with stochastic fluctuations in ion channels (Diba et al., 2004, P10).

15. Fig 3 a,b need to be right next to fig 2 b,c to facilitate direct visual comparison between CA1 and DG. This can be achieved by merging figs 2 and 3. **We changed the figure (Figure 2 and 3) layout to accommodate this suggestion. We believe merging the two figures would make the figures very dense and hard to follow.

16. Tick/axis labels and keys within many figures have very small fonts. These should be increased to match those used in Fig 2a. **Fixed in all figures.

Reviewer #2 In this manuscript, Kühn and colleagues investigate the application of the fooof / spectral parameterization algorithm to rodent hippocampus data. In doing so, they explore a series of changes / additions to the algorithm, including adding additional iterations and bounds for estimate the periodic components, and adding additional model forms for estimating the aperiodic component. With this updated approach, they investigate data recorded from rodent hippocampus, showing that these updated approaches assist with better fitting such data. They compare the empirical fits across the examined areas and note some differences, in particular in the aperiodic components, between areas CA1 and DG. Overall, I find this manuscript addresses an important question of how best to characterize LFP data as recorded from animal models and provides some useful updates and additions to methods for doing so while motivating these changes with empirical examples. In the current version, I do find there are currently some limitations to some of the descriptions in the manuscript that need addressing before this paper is ready for publication.

Comments 1. There are some limitations to the methods reporting, including that Figure 3 shows power spectra from cortex, but this is not otherwise described in the paper. If cortex recordings are included, this should be described in the methods. Where in the cortex are these recordings from? Are cortex recordings from the same tetrodes before they were lowered into the hippocampus, or some other set of electrodes? **Added in Materials and Methods (Electrophysiology and neural signal acquisition, P3) section now.

2. It is not clear how the component specific errors are computed. The methods report that {aperiodic, periodic} error is computed as the mean absolute difference between "data and the {aperiodic / periodic} fits". Is this with the full data? This doesn't make sense - the error between the aperiodic-only model fit component and the full data would be dominated by the peaks and (due to different peaks across different spectra) this is not comparable across spectra. Perhaps the computation is between the model component and the isolated data components (e.g. ap_data = full spectrum - pe_model). If so, this needs to be clarified, and further discussed, including a note of the properties and limitations of such an approach - perhaps most notably that (if it is indeed using the isolated data components), such a measure is not actually necessarily specific to the component under study in that the 'ap_data' may be dissimilar from the 'ap_model' either because the 'ap_model' is a bad description of the aperiodic component specifically and/or because the 'pe_model' is a bad description of the periodic component, and due to this 'ap_data' is not actually a good estimate of the aperiodic component, regardless of 'ap_model'. Note that the original spectral parameterization method does not describe or use any such component specific metrics (likely due to these issues) and so if developing and applying new metrics here, the paper needs to describe, motivate, and validate them. **Clarified in Materials and Methods (Error estimation, P6) now In brief, the periodic errors are very similar to as done in the original FOOOF.

For the aperiodic errors, we do take the difference between the whole spectrum and the aperiodic fits. Though these errors might not be comparable across spectra, they serve as a good proxy to compare different aperiodic fits for the same spectrum. Thus, we apply paired t-tests for this comparison to highlight that for each spectrum these errors get significantly better with our fittings (Figure 3, 3-1, 4).

3. The paper uses some simulations to evaluate the changes to the fitting method, which is an important way to demonstrated and validate the methodological changes. However, the simulations appear very limited - it would appear the simulations only test high powered peaks with a specific exponent and no noise? If so, such a limited range of simulations is not adequate to make the general claim, as is stated, that these modifications improve periodic component fitting in the way they do, without noting the specificity of the simulations and test cases examined. If the claim is that the change in methods is general, a much broader range of simulations is required, as it is not clear that the finding in this specific simulation regime generalizes (note that the original fooof method is targeted at addressing a broad range of potential spectra, as exemplified by the broad range of simulations explored in the paper). In the context of this paper, it would also seem appropriate to note that the goal is specifically testing a specific context of the pattern seem in the empirical data under study, but then the claims made about the performance need to be appropriately discussed in terms of the scope of this demonstration, and the generalizability (or potentially, lack thereof) should be noted. **As mentioned in the methods, our simulations are meant to emulate tetrode ephys hippocampal signals. To do this, we maintain the amplitude of the noise levels in the simulated data the same as real data, as is also evident in the new Figure 1a now. Further, we simulate both the peaks that are most prominent in rodent hippocampus, namely theta and gamma, with the same power, center frequency and bandwidth as in most (median) of the real data. We also tested a wide range of exponents. In Figure 1, we show the trends with only one exponent (i.e. 1.2, reasoning explained in the methods), but we also show the evolution with exponents in Figure 1-1c. Thus, we do not think our simulations are limited, and we do believe our claims from Figure 1 can be generalized to hippocampal ephys signals.

4. In addition, there are some aspects of the figure presentation that are a bit unclear and could perhaps be ameliorated:

- In Figure 1 it would be useful to put titles on the panels to clarify what is being plotted - The organization of results across figures is a little unclear. Fig 2BC and Figure 3AB are (I think?) the same analysis for CA1 and DG respectively? It's unclear why these are split across different figures. This also seems inconsistent with the figure titles - Figure 3 is described as a comparison between CA1 &DG, but 3AB is not a comparison between regions. **Changed the figure (Figure 2 and 3) formats to accommodate these suggestions.

- Figure S1 incorrectly uses a sequential color map for data that crosses 0 (A, B) and should therefore us a diverging colormap **For aesthetic comparisons, we use the same colormap for Figure 1-1 as Figure 1. The color bars are clearly labeled with corresponding values for each figure.

5. The paper notes the previous literature linking measures of the aperiodic exponent to the E/I relationship, in particular from the Gao, 2017 paper. At times, notably at the end of the discussion, it seems to imply that this interpretation from the Lorentzian model of that paper can be applied to the results in the current paper, which use different model forms. It is not one clear can take the findings of Gao, 2017 with one particular model, and apply it to a different model form, as the measured parameters are not interchangeable and the way they relate to underlying physiology may differ. For example, how does one map the exponent of the Lorentzian knee model to the exponents of, for example, the 3exp model in DG? If the E/I link is to be evoked for the new empirical results here, if and how this interpretation relates to the prior work requires at least some additional discussion. ** We have now modified the results and discussion to highlight these limitations and better link our work with previous studies (P10).

Please also see general comments above.

6. The introduction claims there are only two reports of rodent hippocampus - how was this determined? This doesn't seem to include for that the Gao, 2017 paper examined rodent CA1 (this is noted later in the paper), and seems likely to be an undercount. **Changed it to 'very few' and added Gao, 2017 to these references (P2).

7. Methods: How much data was analyzed? The number of epochs / length of time of data that was included need to be reported in the methods. **Added in Methods (Preprocessing of neural data, P3) section now.

8. Across the manuscript, there are some phrasings or descriptions relating to spectral parameterization that I find hard to understand in terms of what they specifically are meant to refer to, and in some cases seem may misreport details of the previous methods which require clarification and/or correction:

- Introduction: What does aperiodic parameters are 'multi-fold' mean? - Methods / Simulated data section: what does "heighten the fallacies of FOOOF" mean / refer to? - Methods / Detection of periodic components: "This modification is different from the 'Band-by-Band' approach introduced with FOOOF 2.0, namely specparam, which relies on removing unwanted Gaussian peaks in post-analysis." It is unclear what this refers to? - The manuscript describes the fooof single exponent model as "linear fitting". This is incorrect - while the model is equivalent to the linear fit of log-log power spectrum, this is not what the fitting process is, which fits 1/f functions in semi-log space. **Modified the original sentences for all of the above concerns (P1, P4, P6, P7, respectively).
