# Supplementary figures and images for: Aperiodicity in Mouse CA1 and DG Power Spectra
Source: eNeuro. 2026 Mar 25;13(3):ENEURO.0136-25.2026. doi: 10.1523/ENEURO.0136-25.2026 (PMC13064425; doi:10.1523/ENEURO.0136-25.2026)

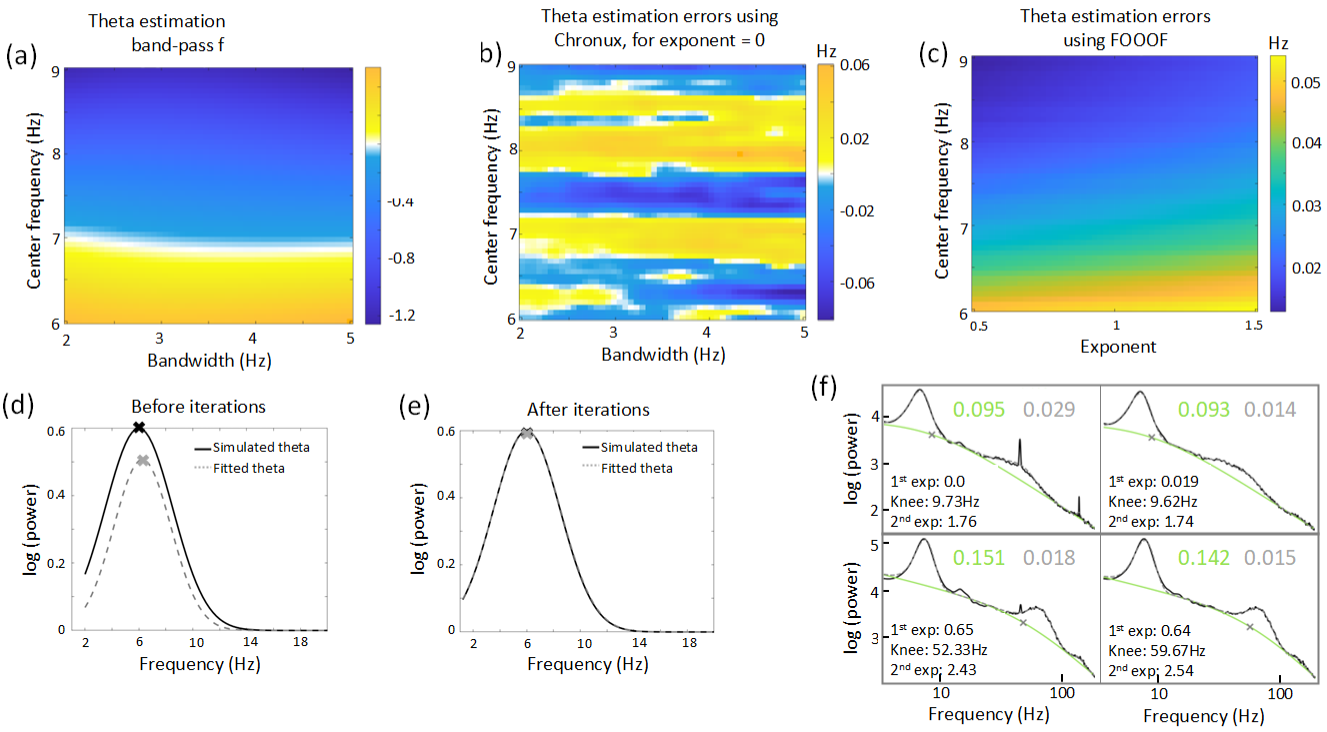

Supplement: Figure 1-1 — Better estimates of the periodic components in simulated electrophysiological signals (a) Errors in cf estimates of the simulated theta peak with pink-noise exponent of 1.2, using band-pass filtering. (b) Errors in cf estimates of the simulated theta peak with pink-noise exponent of 0, using Chronux. This was smoothed using a Gaussian filter with std of 1. (c) Average errors in cf estimates using FOOOF, of the simulated theta peak (with bw of 3.5 Hz), with varying exponent and cf. (d) Flattened simulated and assessed theta Gaussian peak (cf = 6 Hz, bw = 5 Hz, exponent = 1.2) using FOOOF, without iterations. (e) Same as (d) but after 20 iterations. (f) Removal of noise peaks during preprocessing does not significantly affect the estimated aperiodic parameters. Download Figure 1-1, TIF file. [file eneuro-13-ENEURO.0136-25.2026-s002.tif]

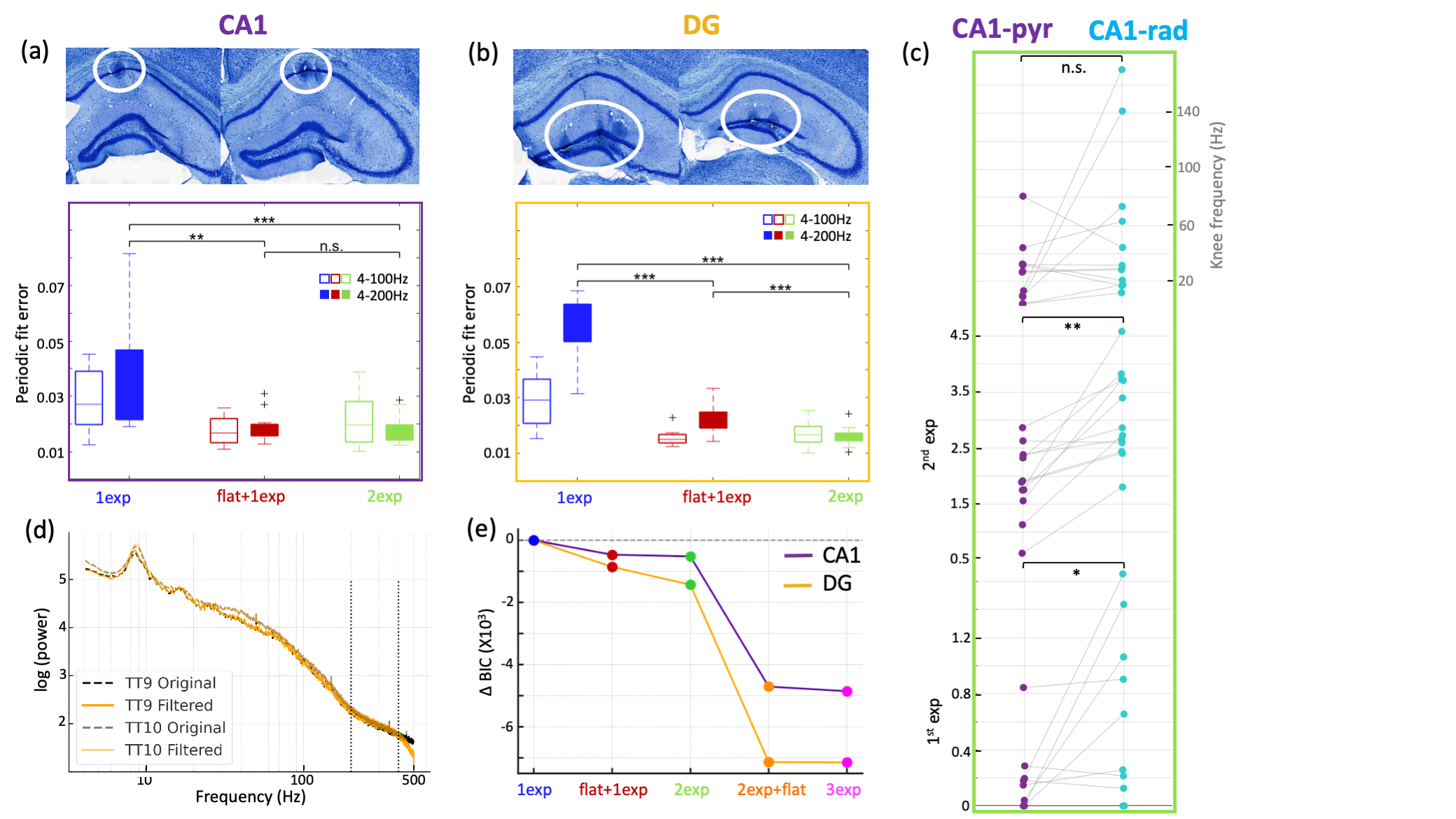

Supplement: Figure 3-1 — Further quantification of our model fittings (a)(top) Tetrode sites were confirmed post-mortem with histology (bottom). Full model fit errors for CA1 signals across ‘1exp’, ‘flat+1exp’ and ‘2exp’, when fit in the ranges of 4-100 Hz (open boxes) and 4-200 Hz (filled boxes). 4-200 Hz paired t-test, p(‘1exp’|’flat + 1exp’) = 5.33X10−4, p(‘1exp’|’2exp’) = 3.31X10−4, p(‘flat+1exp’|’2exp’) = 0.0547. (b) Same as (a) but for DG. (bottom) 4-200 Hz paired t-test, p(‘1exp’|’flat + 1exp’) = 2.97X10−9, p(‘1exp’|’2exp’) = 3.98X10−11, p(‘flat+1exp’|’2exp’) = 6.98X10−6. (c) Change in the aperiodic components for CA1-pyr and CA1-rad signals using ‘2exp’ fitting. Paired t-test, 2nd exponent: p = 7.62X10−4, 1st exponent: p = 0.0473, ‘knee’ frequency: p(‘flat+1exp’|’2exp’) = 0.135. (d) psds of 2 example DG signals, original and, after filtering and down-sampling. Dashed vertical lines at 200 Hz and 400 Hz. (e) The change in BIC scores across the 5 aperiodic fitting models, averaged over all CA1 and DG signals. For all figures, *** p < 0.0005, ** p < 0.005, * p < 0.05, n.s. not significant. Download Figure 3-1, TIF file. [file eneuro-13-ENEURO.0136-25.2026-s003.tif]
